# *Plasmodium* actin is incompletely folded by heterologous protein-folding machinery and likely requires the native *Plasmodium* chaperonin complex to enter a mature functional state

**DOI:** 10.1096/fj.15-276618

**Published:** 2015-10-06

**Authors:** Maya A. Olshina, Hella Baumann, Keith R. Willison, Jake Baum

**Affiliations:** *Walter and Eliza Hall Institute of Medical Research and Department of Medical Biology, University of Melbourne, Parkville, Victoria, Australia; ^†^Department of Life Sciences and ^‡^Department of Chemistry, Imperial College London, South Kensington, London, United Kingdom

**Keywords:** malaria, apicomplexa, gliding motility, *in vitro* translation, rabbit reticulocyte lysate

## Abstract

Actin filament turnover underpins several processes in the life cycle of the malaria parasite, *Plasmodium falciparum*. Polymerization and depolymerization are especially important for gliding motility, a substrate-dependent form of cell movement that underpins the protozoan parasite’s ability to disseminate and invade host cells. To date, given difficulties in extraction of native actins directly from parasites, much of our biochemical understanding of malarial actin has instead relied on recombinant protein extracted and purified from heterologous protein expression systems. Here, using *in vitro* transcription-translation methodologies and quantitative protein-binding assays, we explored the folding state of heterologously expressed *P. falciparum* actin 1 (PfACTI) with the aim of assessing the reliability of current recombinant-protein-based data. We demonstrate that PfACTI, when expressed in non-native systems, is capable of binding to and release from bacterial, yeast, and mammalian chaperonin complexes but appears to be incompletely folded. Characterization of the native *Plasmodium* folding machinery *in silico*, the chaperonin containing t-complex protein-1 complex, highlights key divergences between the different chaperonin systems that likely underpins this incomplete folded state. These results highlight the importance of characterizing actin’s folded state and raise concerns about the interpretation of actin polymerization kinetics based solely on protein derived from heterologous expression systems.—Olshina, M. A., Baumann, H., Willison, K. R., Baum, J. *Plasmodium* actin is incompletely folded by heterologous protein-folding machinery and likely requires the native *Plasmodium* chaperonin complex to enter a mature functional state.

Apicomplexan parasites are an ancient phylum of obligate intracellular protozoa that includes some of the most significant pathogens affecting global human populations (1). The phylum includes *Toxoplasma gondii*, *Cryptosporidium* spp., and parasites from the genus *Plasmodium,* the causative agent of human malaria disease. Cell movement in these parasites, also referred to as gliding motility, underpins the ability of parasites to infect host tissues and invade host cells and is dependent on the linkage of an intracellular parasite myosin motor with dynamic filaments of actin (2). This actomyosin system provides a rearward propulsive force that, when linked to extracellular substrates, drive parasites forward or into host cells (2).

*Plasmodium* parasites express two actin isoforms, ACTI and ACTII, that are markedly divergent from their eukaryotic counterparts (3). ACTI is conserved between *Plasmodium* and other apicomplexan parasites (4) and is the isoform that is directly implicated in gliding motility (5, 6). However, despite the importance of ACTI in cell motility, actin filaments are not readily detected in parasites using various microscopy techniques (7, 8). This is explained by both the reported dominance of globular (G) over filamentous (F) actin in parasite cells (4, 9) and several unusual properties reported for ACTI filaments; namely, their propensity to form very short polymers, measuring only ∼100 nm in length and their highly dynamic, transient, and unstable nature (10, 11). *In vivo* visualization of native actin-rich structures is only possible following treatment of cells with an actin filament-stabilizing drug such as jasplakinolide (7, 12, 13).

With the exception of those studies using extracted PfACTI (10, 11), most work with ACTI has relied on its expression *via* heterologous systems (namely yeast and insect cells) to purify enough protein for assaying (10, 11, 14–17). *Plasmodium falciparum* ACT1 (PfACTI), or that from *Toxoplasma gondii* (TgACT1), expressed in these conditions only, forms long filaments when assayed in the presence of jasplakinolide or equimolar concentrations of phalloidin, another F-actin binding drug. This supports the observation that native apicomplexan actin forms unstable filaments, which precludes extended elongation (16, 18). Indeed, in support of this, when sites divergent to mammalian actin are mutated to more canonical states in TgACTI, this gives much greater filament stability to actin polymers in *Toxoplasma* parasites, permitting the formation of long structures that severely disrupt normal cell function (16).

Alongside *Toxoplasma* work, crystal structures of recombinant *Plasmodium* PfACTI suggests the instability of ACTI filaments is based on the divergence of several key amino acids, in particular the DNaseI binding-loop (D-loop) and the C terminus, which likely function in maintaining intrastrand contacts (18). In PfACTI, the D-loop is disordered and partially missing in the electron density maps, likely affecting the lateral contacts within the filament and therefore overall filament stability (18). When it is replaced with a canonical D-loop, this restores long filament formation *in vitro*. The same study revealed that the C terminus of PfACTI is also disordered and unstructured (18) (see Discussion). This latter result is striking given that correct placement of the C terminus is known to be crucial for native G-actin folding (19). Of note, in the PfACTI–D-loop chimera, the C terminus forms a more native order (18). The reappearance of order in this unrelated domain may question whether disorder is actually the native ACTI state or whether the lack of structure at the C terminus is in fact a product of folding in heterologous expression systems.

In eukaryotic cells, actin is folded into its mature, native form by the *t*-complex protein-1 (TCP-1) ring complex or chaperonin containing TCP-1 (CCT) complex, a 1-MDa protein complex comprised of 8 different subunits arranged into 2 asymmetric rings (20). Actin folding by the CCT complex is an ATP-dependent, multistage process regulated by cofactors from the phosducin-like protein family of proteins. The ability of the CCT complex to fold actin is isoform and species specific, with amino acid divergences leading to incompatibilities between certain actin isoforms and CCTs from different species (21). Given the demonstrated incompatibilities between CCT and heterologous actins, it is possible that the various apicomplexan ACTI expressed proteins may be arrested in intermediate folding states due to incompatibility with the host chaperonin complex.

Here, we sought to address this question using prokaryotic and eukaryotic *in vitro* transcription and translation systems coupled with native PAGE analysis. We demonstrate that PfACTI and human β-actin expressed in prokaryotic expression systems are not functional and cannot interact with a eukaryote CCT complex in the absence of other cofactors. Furthermore, we show that PfACTI expressed in a mammalian eukaryotic expression system, despite being able to bind and be released by CCT, is incompletely folded by heterologous chaperonin systems. To explore the molecular basis for this incompatibility, we identify the presence of an entity isolated from parasite cell lysate that, in being able to bind actin, is consistent with their being a native PfCCT complex. We back this up by identifying orthologous genes for the CCT complex in the *P. falciparum* genome. *In silico* analysis of these identified subunits highlights key divergent residues that likely underpin their incomplete protein folding by non-native CCT. These observations suggest that functional PfACTI likely requires homologous CCT to complete its native monomer fold.

## MATERIALS AND METHODS

### *In vitro* translation and analysis

#### *In vitro* translation

Human β-actin cDNA, in the pET-11d vector (22), and pET-28b-PfACTI were translated *in vitro* using the Expressway Cell-Free *Escherichia coli* Expression System (Life Technologies, Carlsbad, CA, USA) or the T_N_T Coupled Reticulocyte Lysate Systems (Promega, Madison, WI, USA) according to the manufacturers’ instructions. Both systems were coupled with the inclusion of [^35^S]methionine (>1000 Ci/mmol; PerkinElmer, Waltham, MA, USA). Aliquots were taken at appropriate time points and stored on ice until the conclusion of the time course. For the titration experiments, [^35^S]PfACTI, expressed in either the prokaryotic or eukaryotic lysate for 40 min, was added to *P. falciparum* cell extract at a volume ratio 1:10 (*in vitro* translation lysate: *P. falciparum* lysate) and incubated for another 15 min. The *P. falciparum* cell extract was prepared from saponin-lysed parasite pellets, lysed *via* bead-beating after resuspension in extract preparation buffer [45 mM 4-(2-hydroxyethyl)-1-piperazineethanesulfonic acid, 250 mM sucrose, 100 mM KOAc, 2.5 mM Mg(OAc)_2_, and 2 mM DTT] prior to a 10-min centrifugation (12,000 *g*; 4°C) to remove cell debris. Samples were adjusted with 4× Native PAGE Sample Buffer (Life Technologies) and separated on 3–12% Native PAGE Bis-Tris Protein gels (Life Technologies) according to the manufacturer’s instructions, incorporating NativeMark Unstained Protein Standard (Life Technologies). Native PAGE running buffers (Life Technologies) were supplemented with 1 mM ATP.

#### Autoradiography

Native PAGE gels were incubated in 100% v/v DMSO for 20 min at room temperature on an orbital shaker. The gel was incubated in fresh 100% (v/v) DMSO for 20 min, followed by incubation in 22% (w/v) 2,5-diphenyloxazole in DMSO overnight. The gels were washed multiple times in fresh, deionized water until all precipitated residue had been removed. The gels were dried onto Whatman filter paper (GE Healthcare, Little Chalfont, United Kingdom) using a Bio-Rad Gel Dryer 583. ^35^S-Labeled proteins were visualized by autoradiography using BioMax MR film (Bio-Rad, Hercules, CA, USA) or a Fuji FLA-5000 PhosphorImager (Fujifilm, Tokyo, Japan). Band intensities were quantified using AIDA software (FinalWire, Budapest, Hungary), and images were analyzed and processed using ImageJ (U.S. National Institues of Health, Bethesda, MD, USA).

### Protein expression and/or purification

#### Yeast CCT

Calmodulin-binding peptide tagged *Saccharomyces cerevisiae* CCT was expressed and purified as previously described (23). Briefly, cells were lysed, and crude extract was collected and incubated with calmodulin resin (Stratagene, Cedar Creek, Texas, USA). The protein was eluted and subjected to 2 sucrose gradients, followed by concentration of the CCT-containing fractions. The concentrated sample was applied to a 20 ml Superose 6 gel-filtration column (10/300 GL), and the CCT was peak concentrated, adjusted to 50% glycerol, and flash frozen in liquid N_2_.

#### PfADF1/HsCof

Full-length *P. falciparum* actin depolymerizing factor 1 (PfADF1) and *Homo sapiens* cofilin (HsCof) were expressed and purified as N-terminal glutathione *S*-transferase fusion proteins using the pGEX4T vector (GE Healthcare) as previously described (24). Briefly, GST-PfADF1 and GST-HsCof were expressed in BL21 (DE3) *E. coli* cells on addition of 1 mM isopropyl β-d-1-thiogalactopyranoside for 4 h at 37°C, and the cells were harvested, resuspended in lysis buffer (20 mM Tris, pH 8.0, 0.3 M NaCl, 0.3% Triton X-100, and 5 mM 2-mercaptoethanol) supplemented with complete EDTA-free protease inhibitors and 1 mg/ml lysozyme, and lysed by sonication. The proteins were bound to glutathione agarose (Sigma-Aldrich, St. Louis, MO, USA), washed with 100 ml wash buffer 1 (20 mM Tris, pH 8.0, 1 M NaCl, 2% Triton X-100, and 5 mM 2-mercaptoethanol) and 100 ml wash buffer 2 (20 mM Tris, pH 8.0, 1 M NaCl, and 5 mM 2-mercaptoethanol), and eluted with elution buffer (20 mM Tris, pH 8.0, 150 mM NaCl, 20 mM glutathione, and 5 mM 2-mercaptoethanol). The eluted proteins were dialyzed against PBS, and the GST tag was removed by thrombin protease. The cleaved proteins were subjected to size exclusion chromatography on a Superdex 200 10/300 gel filtration column (GE Healthcare) pre-equilibrated in storage buffer (20 mM 2-(*N*-morpholino)ethanesulfonic acid, pH 7.0, and 10 mM NaCl). PfADF1 eluted at ∼18 ml. HsCof eluted at ∼17 ml. The purity of the proteins was assessed by Coomassie Brilliant Blue-stained SDS-PAGE, with positive fractions concentrated and the proteins stored at 4**°**C.

#### PfProfilin

Full-length *P. falciparum* profilin (PfPfn) was expressed and purified as an N-terminal 6xHis fusion protein using the pProEX-HTb (Invitrogen, Carlsbad, CA, USA) as previously described (25). In brief, His-PfPfn was expressed in BL21 (DE3) *E. coli* cells on addition of 1 mM IPTG for 3 h at 37**°**C. The cells were harvested, resuspended in lysis buffer (20 mM Tris, pH 8.0, 150 mM NaCl, 0.3% Triton X-100, 20 mM imidazole, pH 8.0, and 5 mM 2-mercaptoethanol) supplemented with complete EDTA-free protease inhibitors, 1 mg/ml lysozyme, and 1 mg/ml DNAseI. The suspension was incubated on a rolling platform at 4**°**C for 30 min, followed by centrifugation at 30,000 *g* for 30 min. The supernatant was collected and incubated for 2 h at 4**°**C with 4 ml Profinity IMAC resin (Bio-Rad). The resin was washed sequentially with wash buffer 1 (20 mM Tris, pH 8.0, 150 mM NaCl, 2% Triton X-100, 20 mM imidazole, pH 8.0, and 5 mM 2-mercaptoethanol) and wash buffer 2 (20 mM Tris, pH 8.0, 150 mM NaCl, 20 mM imidazole, pH 8.0, and 5 mM 2-mercaptoethanol) and eluted with elution buffer (20 mM Tris, pH 8.0, 150 mM NaCl, 250 mM imidazole, pH 8.0, and 5 mM β-mercaptoethanol). The protein was dialyzed against Tris-buffered saline (150 mM Tris, pH 8.0, and 150 mM NaCl), and the N-terminal 6×His tag was removed by cleavage with tobacco etch virus protease. The cleaved protein was subjected to size exclusion chromatography on a Superdex 200 10/300 gel filtration column (GE Healthcare) pre-equilibrated in Tris-buffered saline. PfPfn eluted at ∼15.5 ml, and the protein purity was assessed by Coomassie-stained SDS-PAGE. PfPfn was concentrated to 1 mg/ml and stored at 4°C.

### Sequestration assays

Rabbit skeletal muscle actin (2 μM) in Ca-buffer G (2 mM Tris, pH 8.0, 0.2 mM ATP, 0.5 mM DTT, and 0.1 mM CaCl_2_) was induced to polymerize in the presence of PfPfn/PfADF1 (0–16 μM) by the addition of 10× KMEI (0.5 M KCl, 0.1 M imidazole, pH 7.0, 0.01 EGTA, pH 8.0, and 0.01 M MgCl_2_) and incubated for 2 h at room temperature. The samples were centrifuged at 60,000 rpm in a Beckman preparative ultracentrifuge for 1 h at room temperature. The supernatant was carefully removed and adjusted with 5× reduced sample buffer. The pellet was rinsed with MgBG (2 mM Tris, pH 8.0, 0.2 mM ATP, 0.5 mM DTT, and 0.1 mM MgCl_2_) and centrifuged at 60,000 rpm in a Beckman preparative ultracentrifuge for 1 h at room temperature. The supernatant was carefully removed and discarded, and the pellet was resuspended in 2× reduced sample buffer to a volume equivalent to the first supernatant after addition of reduced sample buffer. The supernatant and pellet samples were boiled for 5 min, equal volumes were separated by SDS-PAGE, the gels were stained with Coomassie Brilliant Blue (Bio-Rad), and the bands were analyzed by densitometry (Supplemental Data).

### Computational analyses

#### BLASTp searches and sequence alignments

The sequences of CCT1 from *H. sapiens* and *S. cerevisiae* were used as BLASTp queries to search the *P. falciparum* genome to identity potential homologes of CCT (www.plasmodb.org). Selected *P. falciparum* sequences were used for reciprocal BLASTp queries of the *H. sapiens* (taxid: 9606) and *S. cerevisiae* (taxid: 4932) databases (blast.ncbi.nlm.nih.gov) to confirm their identities. Multiple sequence alignments were generated by ClustalW2 (www.ebi.ac.uk/Tools/msa/clustalw2) and collated using ESPript 3.0 (www.espript.ibcp.fr/ESPript/ESPript).

#### *In silico* modeling

Modeling was performed by submitting protein sequences to the I-TASSER server (www.zhanglab.ccmb.med.umich.edu/I-TASSER) (26). Generated models were analyzed and prepared using PyMOL (DeLano Scientific LLC, Palo Alto, CA, USA; www.pymol.org).

## RESULTS

### PfACTI can be expressed in prokaryotic and eukaryotic *in vitro* translation systems

**Figure 1.**
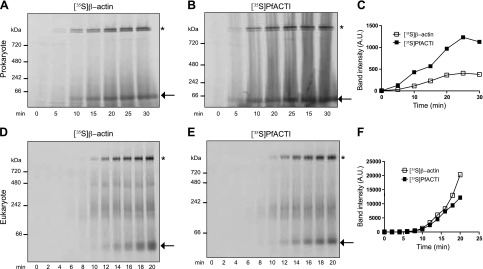
*In vitro* translation of actins in *E. coli* cell extract and rabbit reticulocyte cell extract. *A*, *B*) Time course of expression of [^35^S]β-actin (*A*) and [^35^S]PfACTI (*B*), translated in *E.coli* *in vitro* transcription/translation extract, monitored by native 3–12% PAGE analysis. Arrow, [^35^S]β-actin/PfACTI; asterisk, [^35^S]β-actin/PfACTI bound to endogenous GroEL present in the *E. coli* cell extract. *C*) Densitometry of band intensities over the time course of expression, for both [^35^S]β-actin (open squares) and [^35^S]PfACTI (filled squares). *D*, *E*) Time course of expression of [^35^S]β-actin (*D*) and [^35^S]PfACTI, translated in rabbit reticulocyte *in vitro* transcription/translation extract, monitored by native 3–12% PAGE analysis (*E*). Arrow, monomeric [^35^S]β-actin and [^35^S]PfACTI; asterisk, [^35^S]β-actin and [^35^S]PfACTI bound to endogenous RbCCT. *F*) Densitometry of the monomeric actin band intensities over the time course of expression, for both [^35^S]β-actin (open squares) and [^35^S]PfACTI (filled squares).

Well-established methods for analyzing the folding state of actin monomers incorporating *in vitro* transcription and translation coupled with ^35^S labeling, native PAGE analysis and autoradiography have been used extensively to characterize the folding kinetics of canonical actins, such as human β-actin (β-actin) and yeast ACT1 (23). To determine whether PfACTI can be expressed using the same systems, a time course of protein expression was performed using prokaryotic (*E. coli*) and eukaryotic (rabbit reticulocyte) cell lysate expression systems. It has been well established that actin expressed in *E. coli* is not functional; therefore, this system was included alongside the eukaryotic expression system as a negative control. Time courses for human β-actin expression were run in parallel. Both [^35^S]β-actin (Fig. 1*A, D*) and [^35^S]PfACTI (Fig. 1*B, E*) were translated efficiently in both systems, with an increase in signal in a discrete band seen below the 66-kDa marker. Quantification of band intensity by densitometry demonstrates the increase in protein expression over time for both actins (Fig. 1*C, F*). Although there does appear to be a higher level of expression of [^35^S]PfACTI compared with [^35^S]β-actin in the prokaryotic expression system (Fig. 1*C*), this could be a result of slight variation in DNA template concentration, which is amplified during RNA transcription and translation.

In addition to the band representing monomeric actin in each experiment, there are higher-molecular-weight bands appearing toward the top of the gels above the 720-kDa marker, as indicated by the asterisks (Fig. 1*A, B, D, E*). It has been previously reported that nascent, unfolded actin can form a tight complex with GroEL, the primary chaperonin present in bacterial cells, although GroEL cannot process actin into its native state (27). This higher banding in the prokaryotic expression system therefore denotes the complex formed by endogenous *E. coli* GroEL and the translated [^35^S]β-actin or [^35^S]PfACTI (Fig. 1*A, B*). In the eukaryotic system, however, the discrete, higher-molecular-weight band appearing toward the top of the gels represents the complex formed between incompletely folded [^35^S]β-actin or [^35^S]PfACTI and endogenous rabbit CCT (RbCCT), which is present in the rabbit reticulocyte cell extract (Fig. 1*D, E*). This banding pattern for CCT bound to actin has been validated in multiple studies in both yeast and rabbit expression systems (23). This indicates that [^35^S]PfACTI can bind to RbCCT, although whether it is being processed into its native, folded state is not clear.

### PfACTI expressed in prokaryotic cells can bind to the CCT complex in the eukaryotic cell lysate

**Figure 2.**
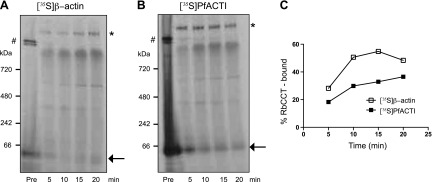
Competition of *E. coli* extract *in vitro*-translated actins onto endogenous rabbit CCT. *A*, *B*) [^35^S]β-Actin (*A*) and [^35^S]PfACTI (*B*), translated in *E. coli* cell extract for 30 min (Pre), added to rabbit reticulocyte extract and monitored by native 3–12% PAGE analysis for 20 min. Arrow, [^35^S]β-actin/PfACTI; asterisk, [^35^S]β-actin/PfACTI bound to endogenous CCT present in the rabbit reticulocyte cell extract; #, [^35^S]β-actin/PfACTI bound to GroEL. *C*) Quantification by densitometry of the percentage of actin bound to RbCCT over time for both [^35^S]β-actin (open squares) and [^35^S]PfACTI (filled squares).

To determine whether the actins expressed in the prokaryotic system are capable of binding to RbCCT in the eukaryotic cell extract, [^35^S]β-actin and [^35^S]PfACTI were expressed for 30 min in the prokaryotic *in vitro* translation system followed by titration into the eukaryotic cell extract. Both [^35^S]β-actin and [^35^S]PfACTI bind to RbCCT, as denoted by the change in banding pattern before and after titration (Fig. 2*A, B*). As the time course progresses, the RbCCT-actin band increases in intensity for both [^35^S]β-actin and [^35^S]PfACTI, whereas the GroEL-actin complex band disappears, indicating total competition of the actins off GroEL and onto RbCCT. Quantification of the relative percentage of [^35^S]β-actin or [^35^S]PfACTI bound to RbCCT at each time point demonstrates this increase in RbCCT binding (Fig. 2*C*).

### PfACTI expressed in eukaryotic cell lysate can bind to *S. cerevisiae* CCT

**Figure 3.**
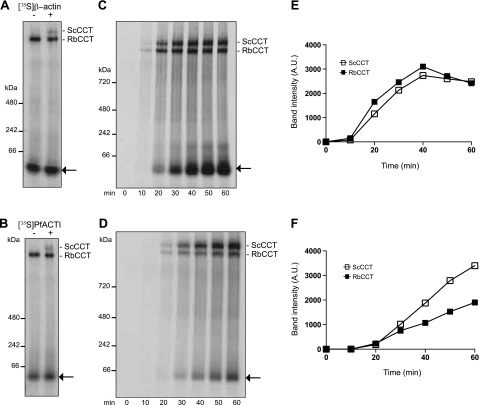
Rabbit reticulocyte actins can bind to ScCCT. Native PAGE analysis of [^35^S]β-actin and [^35^S]PfACTI translated in rabbit reticulocyte extract in the presence of purified ScCCT (400 nM). *A*, *B*) [^35^S]β-Actin (*A*) or [^35^S]PfACTI (*B*) was expressed for 40 min (left lane), followed by addition of ScCCT (right lane). [^35^S]β-Actin/[^35^S]PfACTI bound to ScCCT runs higher that [^35^S]β-actin/[^35^S]PfACTI bound to endogenous RbCCT as indicated by ScCCT/RbCCT labels. *C*, *D*) [^35^S]β-Actin (*C*) and [^35^S]PfACTI (*D*) were translated in the presence of purified ScCCT (400 nM) for 60 min with samples taken every 10 min. Arrow indicates translated [^35^S]β-actin/[^35^S]PfACTI. *E*, *F*) Quantification of band intensities over the time course of expression for [^35^S]β-actin (*E*) and [^35^S]PfACTI (*F*) bound to ScCCT (open squares) and RbCCT (filled squares).

To explore the affinity of PfACTI for an alternative eukaryotic CCT complex, we next determined whether it could interact with that from *S. cerevisiae.* Purified *S. cerevisiae* CCT (ScCCT) (23) was added to the rabbit reticulocyte *in vitro* translation system following [^35^S]β-actin and [^35^S]PfACTI expression, thus discriminating between actin binding to endogenous RbCCT over the added ScCCT (Fig. 3*A, B*). Comparison of the banding pattern at the top of the gels indicates that the band corresponding to ScCCT bound to either [^35^S]β-actin (Fig. 3*A*) or [^35^S]PfACTI (Fig. 3*B*) runs slightly slower than endogenous RbCCT-actin. To compare the binding efficiency for RbCCT and ScCCT for each actin over the course of translation, ScCCT was added at the initiation of translation, with samples taken every 10 min for 60 min (Fig. 3*C, D*). [^35^S]β-Actin binds to both endogenous RbCCT and added ScCCT with approximately the same efficiency (Fig. 3*C*) with further analysis by densitometry confirming this similarity (Fig. 3*E*). In contrast, [^35^S]PfACTI shows a distinct preference for binding to ScCCT (Fig. 3*D*). Analysis by densitometry clearly demonstrates this effect, with a large proportion of [^35^S]PfACTI partitioning to ScCCT (Fig. 3*F*). It is also important to note that [^35^S]β-actin peaks in binding to both CCTs at 40 min and then begins to show release from the CCTs (Fig. 3*E*), whereas [^35^S]PfACTI continues to accumulate on the CCTs and does not show increased release (Fig. 3*F*). This explains why there appears to be a higher yield of unbound [^35^S]β-actin compared with [^35^S]PfACTI (indicated by the arrows, Fig. 3*C, D*), as native [^35^S]β-actin is released from CCT, whereas [^35^S]PfACTI is not. These data hint toward complications in the completion of the folding cycle by CCT for PfACTI, which should release actin once it is natively folded (28).

### PfACTI expressed in eukaryotic cell lysate is unable to bind to G-actin binding proteins and is therefore not natively folded

Although PfACTI is able to bind to different eukaryotic CCTs, ScCCT and RbCCT, it is unclear whether even a small proportion is being processed by CCT and folded into its native state. A method of determining the formation of natively folded actin in the context of *in vitro* translation is the ability of added G-actin binding proteins to interact with monomeric actin (23). If β-actin is natively folded, it will be able to form a complex with the added G-actin binding protein DNAseI, forming a complex that will be visible as a higher-molecular-weight band after native PAGE analysis (29). However, sequence analysis and experimental evidence have suggested that the DNAseI binding site on PfACTI is highly divergent, leading to a 200-fold reduction in DNAseI binding affinity (15). Therefore, although DNAseI is an appropriate choice to assess the native fold of β-actin, 2 additional G-actin–binding proteins, PfADF1 (24, 30) and PfPfn (25), were used for analysis of PfACTI. These proteins have been previously validated as functional G-actin interacting proteins when expressed recombinantly in *E. coli* (25, 30) and were subjected to rabbit skeletal muscle actin sequestration assays prior to use to ensure G-actin binding activity (Supplemental Fig. S1). To mirror the use of alternative G-actin binding proteins, HsCof (24) was used alongside DNAseI for assessment of β-actin folding.

**Figure 4.**
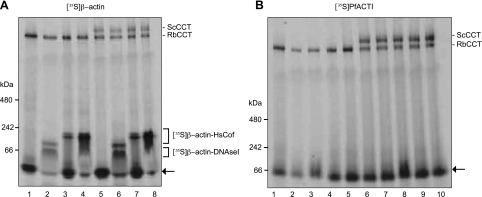
Assessment of native folding of actins by complex formation with G-actin binding proteins. Native 3–12% PAGE analysis of actins translated in rabbit reticulocyte extract in the absence or presence of purified ScCCT (400 nM) for 40 min followed by addition of G-actin binding proteins for 15 min. *A*) Lanes 1 and 5, [^35^S]β-actin prior to G-actin binding protein addition synthesized in the presence or absence of ScCCT, respectively; lanes 2 and 6, +1 μg DNAseI; lanes 3 and 7, +1 *μ*g HsCof; lanes 4 and 8, +10 μg HsCof. Complexes formed between [^35^S]β-actin and DNAseI/HsC of marked by square brackets. *B*) Lanes 1 and 6, [^35^S]PfACTI prior to G-actin binding protein addition synthesized in the presence or absence of ScCCT, respectively; lanes 2 and 7, +1 μg PfADF1; lanes 3 and 8, +10 μg PfADF1; lanes 4 and 9, +1 *μ*g PfPfn; lanes 5 and 10, +10 μg PfPfn. Arrows, [^35^S]β-actin/[^35^S]PfACTI uncomplexed.

Prior to the addition of the G-actin binding proteins, [^35^S]β-actin and [^35^S]PfACTI were synthesized for 40 min in the eukaryotic *in vitro* translation system with and without ScCCT, which may be able to bind and fold PfACTI if RbCCT cannot. One microgram DNAseI or 1 *μ*g/10 *μ*g of HsCof was added to the [^35^S]β-actin translation reactions (Fig. 4*A*), and 1 *μ*g/10 *μ*g PfADF1 and 1 *μ*g/10 *μ*g PfPfn were added to the [^35^S]PfACTI translation reactions (Fig. 4*B*). [^35^S]β-Actin formed complexes with both DNAseI and HsCof both in the presence and absence of ScCCT (Fig. 4*A*). Although there is still a residual amount of monomeric actin remaining that is not in a complex, a concentration-dependent shift is evident when comparing the amount of [^35^S]β-actin shifting to the complex with 1 or 10 μg added HsCof. This indicates that [^35^S]β-actin is natively folded in the eukaryotic *in vitro* translation system by endogenous RbCCT. In contrast, [^35^S]PfACTI was not able to form complexes with either PfADF1 or PfPfn, regardless of the amount of G-actin binding protein added, or the presence or absence of added ScCCT (Fig. 4*B*). This inability to bind to G-actin binding proteins would appear to indicate that [^35^S]PfACTI is not natively folded when expressed in the heterologous eukaryotic system, and, furthermore, ScCCT is not capable of mediating its folding into a native state. Combined, these data demonstrate that the production of PfACTI in heterologous eukaryotic expression systems is unlikely to yield fully folded native protein. This suggests that evolutionary specificity may have evolved between parasite actin and its native folding machinery as it has with other actin isoforms in other species (21).

### Addition of *in vitro*-translated PfACTI into *P. falciparum* lysate

The inability of PfACTI to fold into its native state in the context of a heterologous expression system indicates a potential requirement for homologous protein folding machinery found within the *Plasmodium* parasite cell. If [^35^S]PfACTI is in a partially unfolded state after expression in an *in vitro* translation system, it may be able to bind to the homologous *P. falciparum* CCT homolog (PfCCT) within *P. falciparum* cell extract and be folded into its native form.

**Figure 5.**
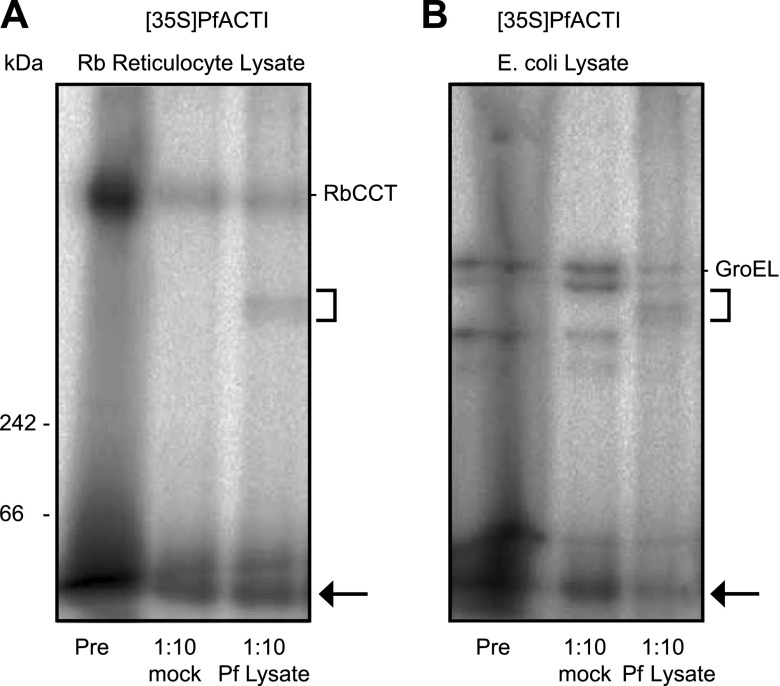
Addition of [^35^S]PfACTI to *P. falciparum* cell extract. Native PAGE analysis of [^35^S]PfACTI translated in rabbit reticulocyte (*A*) or *E. coli* lysate (*B*) for 40 min (Pre), added to extract preparation buffer (mock) or *P. falciparum* cell extract (Pf lysate) at a 1:10 ratio of [^35^S]PfACTI:*P. falciparum* cell extract for 15 min. Square bracket indicates possible complex formation in *P. falciparum* lysate. Arrow, monomeric [^35^S]PfACTI.

To investigate this possibility, [^35^S]PfACTI, expressed in either the eukaryotic or the prokaryotic *in vitro* translation system, was titrated into *P. falciparum* cell extract or the extract preparation buffer as a negative control (Fig. 5 and Supplemental Fig. S2). For the reticulocyte expression system lysate, the band corresponding to [^35^S]PfACTI bound to RbCCT persists after addition to the *P. falciparum* cell extract, however, with a diminished intensity. Concurrently, an additional band, which would be consistent with binding of [^35^S]PfACTI to its native PfCCT present in the lysate, became apparent (Fig. 5*A*). Critically, an identical banding pattern also appeared when the prokaryotic *in vitro* translation lysate was incubated with *Plasmodium* cell extract (Fig. 5*B*). Of particular note, the bands [^35^S]PfACTI bound to GroEL in the prokaryotic cell extract almost entirely disappeared when titrated into the *P. falciparum* cell extracts, supporting the notion that GroEL-bound [^35^S]PfACTI preferentially disengaged to rebind to the native *Plasmodium* CCT complex. These findings demonstrate the presence of a cytosolic *P. falciparum* complex, which can bind to unfolded actin and likely indicates the presence of the PfCCT complex. This would be predicted if, as a eukaryotic cell with a complex cytoskeleton including both actin and tubulin, *Plasmodium* has a conventional homolog of the CCT complex to mediate the folding of cytoskeletal components.

### *In silico* identification of *P. falciparum* CCT

Toward identifying if a CCT complex is present in the *Plasmodium* genome, BLASTp searches were performed on public databases (31) using the protein sequences of CCT1 from *H. sapiens* and *S. cerevisiae*. The reference genome for *P. falciparum* (strain 3D7) yielded 10 CCT subunit hits, of which 2 nonspecific hits could readily be discounted [the 60-kDa chaperonin (CPN60) and heat shock protein 60 (HSP60), both of which have previously been characterized as mitochondrial chaperones (32, 33)]. The remaining 8 genes are all described as putative T-complex protein subunits or members of the TCP-1 chaperonin family. To classify each of the 8 putative genes as subunits of the CCT complex, a reciprocal BLASTp search was performed on the databases of *H. sapiens* (taxid: 9606) and *S. cerevisiae* (taxid: 4932) by submitting the protein sequence for each putative subunit and selecting the top hit. Both the *H. sapiens* and *S. cerevisiae* databases were in agreement regarding the subunit categorization, and all 8 CCT subunits were accounted for. As outlined in Table 1, the percentage identity between the putative *P. falciparum* CCT subunits and those from *H. sapiens* and *S. cerevisiae* were low, ranging from 34% to 60%, indicating a high level of sequence divergence in PfCCT.

**TABLE 1.** Results of CCT subunit protein BLAST search using UniProtKB/SwissProt database

*P. falciparum* gene ID	Description	*H. sapiens* gene ID	% Identity	*S. cerevisiae* gene ID	% Identity	Description
PF3D7_0214000	T-complex protein 1, putative	P50990	34	P47079	34	TCP-1 subunit θ
PF3D7_0306800	T-complex protein β subunit, putative	P78371	57	P39076	55	TCP-1 subunit β
PF3D7_0308200	TCP-1/cpn60 chaperonin family, putative	Q99832	57	P42943	54	TCP-1 subunit η
PF3D7_0320300	T-complex protein 1 ε subunit, putative	P48643	55	P40413	51	TCP-1 subunit ε
PF3D7_0608700	chaperone, putative	P40227	49	P39079	43	TCP-1 subunit ζ
PF3D7_1132200	TCP-1/cpn60 chaperonin family, putative	P17987	59	P12612	55	TCP-1 subunit α
PF3D7_1229500	T-complex protein 1 γ subunit, putative	P49368	54	P39077	51	TCP-1 subunit γ
PF3D7_1357800	TCP-1/cpn60 chaperonin family, putative	P50991	60	P39078	51	TCP-1 subunit δ

### Conservation of signature residues and the nucleotide-binding site in PfCCT

Highly conserved residues, termed “signature residues” have been identified in each CCT subunit across many species (34). These signature residues are located throughout each of the subunits, with those in the apical domains involved in substrate binding, whereas those in the intermediate and equatorial domains are involved in ATP hydrolysis and stability of the complex. To determine whether these residues are conserved in the *P. falciparum* CCT subunits, multiple sequence alignments were generated for each CCT subunit, using protein sequences from *H. sapiens*, *Mus musculus*, *Drosophila melanogaster*, *Caenorhabditis elegans*, *S. cerevisiae*, and *P. falciparum*. Of most interest, in all species analyzed, with the exception of *P. falciparum,* the signature residues are completely conserved (data not shown). In *P. falciparum,* approximately 21% of the signature residues are divergent, with ∼42% of these divergent residues located in the apical domain (Table 2). Given that the apical domain is responsible for substrate binding, this high level of sequence divergence in *P. falciparum* is likely an indication of species-specific interactions with substrates, further lending support to the potential necessity of native CCT for folding PfACTI into a mature state. It is also notable that the CCT-binding sites of mammalian β-actin (35) are divergent in PfACT1, which further strengthens the suggestion of coevolution and divergence of the PfCCT-actin system.

**TABLE 2.** Divergent signature residues in the PfCCT complex

CCT subunit domain	CCT1	CCT2	CCT3	CCT4	CCT5	CCT6	CCT7	CCT8
Equatorial	Q28A*^a^*		D131S			T42S, A77S	P114G	E126D
Intermediate		G186D						G175Y
Apical	L206I, A208V	D209E, A259Q	G255A, P292C	E317D	L340I	M199I, H201R, D223N, V284L, A336P		A292K
Intermediate			S385T, P410G					D402H
Equatorial			T512S	S506T	Q502I	E476K		Y479I

a Residue numbering is given as per ScCCT (34).

Within each subunit of CCT is a conserved cluster of aspartic acid residues, located in the equatorial domain, that are involved in nucleotide binding and ATP hydrolysis. As described for ScCCT6, D58, D84, and D397 coordinate the catalytic water molecule, whereas D89, located within the sequence GDGTT, is the catalytic residue for ATP hydrolysis (34). This sequence is a conserved ATP hydrolysis motif also found in the archaeal thermosome (a homolog of CCT in Archaea) and GroEL (36). To determine whether this nucleotide binding site is conserved in PfCCT, a multiple sequence alignment of all PfCCT subunits was generated, and the aspartic acid residues potentially involved in ATP hydrolysis were identified across the subunits, demonstrating complete conservation of the catalytic site (Supplemental Fig. S3). The motif GDGTT is conserved in subunits PfCCT1-5 and 7. In subunit PfCCT6, the motif is instead GDGSS; however, the latter serines represent somewhat conservative substitutions. PfCCT8 is the most divergent, with the motif consisting of GDFTN. These differences hint toward possible heterogeneity in the ATP hydrolysis potential of the PfCCT subunits as has been demonstrated in ScCCT (36), indicating that PfCCT may act with a similar sequential mechanism for ATP hydrolysis and consequent conformational change during substrate folding.

## DISCUSSION

ACTI is increasingly implicated in diverse processes in the apicomplexan parasite cell, from endocytic trafficking (37, 38) to nuclear positioning of *var* genes associated with parasite virulence (39, 40), gametocyte development (6, 41), cell-cell communication (42), and, of perhaps the most intensive interest, the molecular basis for parasite gliding motility and host cell invasion (43). Gliding motility is known to rely on dynamic actin filaments that are thought to act as a “molecular clutch,” engaging the myosin motor and together generating a rearward force sufficient to drive the parasite forward (2).

Detailed investigation of apicomplexan actin in recent years has demonstrated that it is remarkably different from conventional eukaryotic actins. Indeed, unlike canonical actins, which conform to a nucleation-elongation mechanism of polymerization, it has even been proposed that apicomplexan actins polymerize in a unique fashion, using isodesmic polymerization that involves identical association and dissociation rates throughout polymerization with no lag phase (17). Combined with observations that these actins also form very short, highly dynamic, and unstable polymers (10, 11, 14–16, 18), this has placed apicomplexan actin (ACTI specifically) as one of the most divergent known, both in its sequence and kinetic properties. Some of these properties are thought to be specific evolutionary adaptations of the parasite that allow for the rapid polymer turnover required for invasion and gliding motility (16) and the skewing of the whole actin regulatory system toward disassembly (43).

Of key concern, however, is the paucity of biochemical studies that have focused on native actin from these parasites (10, 11). Most biochemical and kinetic measurements described in the literature have instead relied on purification of ACTI (both *Toxoplasma* and *Plasmodium*) using heterologous yeast or insect cell expression systems (14–16, 18). Here, we set out to test whether heterologous expression of PfACT, the primary actin required for motility in *Plasmodium* parasites, is correctly folded in prokaryotic and eukaryotic coupled transcription/translation systems. This demonstrated that PfACTI produced in both expression systems appears to be incompletely folded into a native, and therefore, fully functional state. This is most keenly demonstrated by the inability of monomeric PfACTI to bind to two of its native G-actin binding proteins (Fig. 4). Although the *in vitro* expression systems used are not identical to those in the published literature, these data certainly raise a critical question surrounding the characterization of PfACTI expressed in either yeast or insect cells.

**Figure 6.**
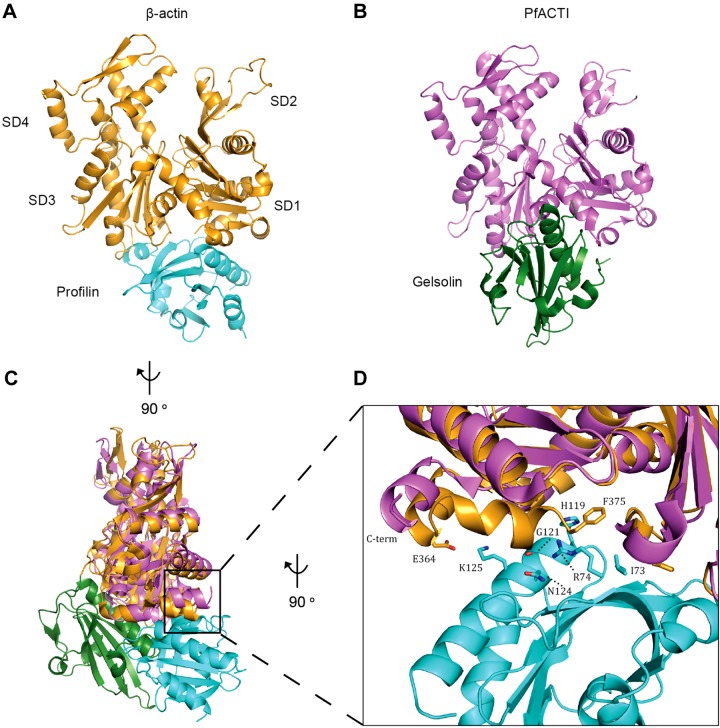
Comparison of structures of β-actin:profilin and PfACTI/gelsolin complexes. *A*, *B*) The crystal structures of β-actin bound to profilin (*A*), where β-actin is in orange and profilin is in cyan [2BTF.pdb (44)] and PfACTI bound to gelsolin (*B*), where PfACTI is in pink and gelsolin is in green [4CBU.pdb (18)]. SDs 1-4 are labeled in *A*. *C*) Overlay of *A* and *B* rotated 90° to the left along the vertical axis, demonstrating the different binding modes of profilin and gelsolin. *D*) Close up view of the interaction between the C terminus of β-actin and profilin, viewed from a 90° rotation to the left along the vertical axis from *C*, overlayed with PfACTI. F375 of β-actin forms contacts with I73, R74, H119, G121, and N124 of profilin, whereas E364 of β-actin interacts with K125 of profilin. Residue side chains are represented as sticks. Note the absence of the C-terminal helix in the structure of PfACTI.

On this point, and of particular interest, is a recent study in which the crystal structure of monomeric PfACTI was solved in complex with the mammalian G-actin binding protein gelsolin (18). Apicomplexan parasites, *Plasmodium* included, do not contain a homolog of gelsolin (43). Close inspection of the PfACTI:gelsolin structure reveals that the last 8 residues, SIVHRKCF, of PfACTI are not visible, indicating disorder at the C terminus (Fig. 6). Of note, the terminal phenylalanine, F375, in human β-actin is critical for binding to profilin (44) (it interacts with I73, R74, H119, G121, and N124 of profilin *via* its phenyl ring and C terminus; Fig. 6*D*). If the C-terminal region is consistently disordered in heterologously, and potentially incompletely folded PfACTI, this might therefore caution interpretation that recombinant PfPfn shows only weak affinity for PfACTI *in vitro* (45).

The contribution of the C terminus to actin function has been extensively examined across many other eukaryotic systems. For example, exposure of the thiol groups in cysteine residues including C374 (the penultimate actin residue), using ATP analogs, results in a loss of actin polymerizability (46). Similarly, direct modification of C374 to glutathionyl, a mixed disulfide, leads to the formation of filaments that are easily disrupted by mechanical stress, indicating that these filaments are more unstable (47). This same study demonstrated that the stability of the filaments formed by this modified actin could be rescued by the addition of equimolar phalloidin (47). The same effect on filament stability can also be achieved by limited proteolysis of the last 2 residues of actin, C374 and F375, which could also be rescued by the addition of equimolar phalloidin (48). In each study, C-terminal truncations have also been shown to increase the critical concentration, polymerization rate, and rate of ATP hydrolysis of the filaments (47–49). C-terminal truncations have also been shown to lead to conformational changes in the d-loop of SD2, disrupting the interactions between neighboring monomers in the filament, affecting filament stability, and causing observed structural changes to the actin filament (49–53). Finally, *in vivo,* truncation of the last 2 residues, C374 and F375, of yeast actin (the last 3 are lethal) leads to a complete loss of actin filamentous structures (54) with a similar effect seen in mammalian cells containing actin having the same mutated C374, which led to a disruption and disorganization of observable filamentous structures with an accompanied increase in diffuse actin staining (55). It is important to note that, from both experimental data (19, 28, 56, 57) and computational modeling (57), the final stage of actin monomer folding *via* CCT is thought to involve the correct positioning of the C terminus in SD1 (19, 28, 56, 57), which is also the final stage that precedes release of native actin from CCT (19, 28). Therefore, although the rest of the protein may be correctly folded, without correct packing of the C terminus in SD1, the protein is not completely folded into its native, and therefore, fully functional, state.

Given this wealth of experimental evidence about the critical role that the C terminus of actin plays in the formation of stable filaments *in vitro* and *in vivo,* the fact that many of the unusual kinetic properties or phenotypic characteristics described above for mutant actins have been ascribed to native apicomplexan actins, based on studies with heterologous expression ACTI, a degree of caution in the interpretation of biochemical assays may now be warranted. Assays with native actin or actin refolded with the native PfCCT complex would now appear to be an imperative.

## Supplementary Material

Supplemental Data

## Abbreviations

ADF: actin depolymerizing factor
CCT: chaperonin-containing TCP-1
Cof: cofilin
d-loop: DNaseI binding loop
Hs: *Homo sapiens*
PfACTI: *Plasmodium falciparum* actin I
PfCCT: *P. falciparum* CCT homolog
Pfn: profilin
RbCCT: rabbit CCT
ScCCT: *S. cerevisiae* CCT
TgACT1: *Toxoplasma gondii* actin 1
TCP-1: t-complex protein-1
