# Supplementary material for: Plasmodium actin is incompletely folded by heterologous protein-folding machinery and likely requires the native Plasmodium chaperonin complex to enter a mature functional state
Source: FASEB J. 2015 Oct 6;30(1):405–16. doi: 10.1096/fj.15-276618 (PMC5423778; doi:10.1096/fj.15-276618)
Supplement: Supplemental Data [file supp_30_1_405__index.html]

Plasmodium actin is incompletely folded by heterologous protein-folding machinery and likely requires the native Plasmodium chaperonin complex to enter a mature functional state — Plasmodium actin is incompletely folded by heterologous protein-folding machinery and likely requires the native Plasmodium chaperonin complex to enter a mature functional state — Supplemental Data 

# *Plasmodium* actin is incompletely folded by heterologous protein-folding machinery and likely requires the native *Plasmodium* chaperonin complex to enter a mature functional state

## Supplemental Data

- Supplemental Data
- Supplemental Data
- Supplemental Data

- Facebook
- Google+
- LinkedIn
- Mendeley
- Reddit
- StumbleUpon
- Twitter

What's this?
