# Supplementary material for: Plasmodium actin is incompletely folded by heterologous protein-folding machinery and likely requires the native Plasmodium chaperonin complex to enter a mature functional state
Source: FASEB J. 2015 Oct 6;30(1):405–16. doi: 10.1096/fj.15-276618 (PMC5423778; doi:10.1096/fj.15-276618)
Supplement: Supplemental Data [file supp_fj.15-276618_Supplemental_Figure1.pdf]

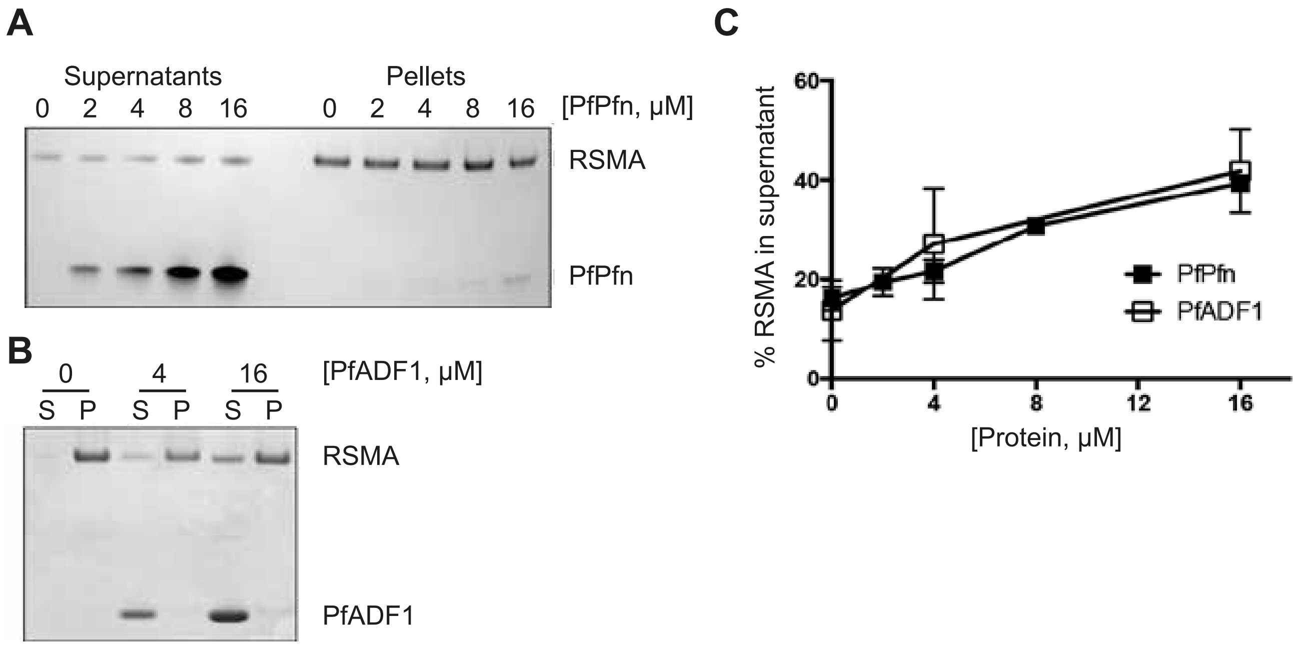

Supplementary Figure 1: RSMA sequestration by PfPfn and PfADF1.
Sedimentation assay demonstrating the G-actin sequestration activity of PfPfn and PfADF1 on RSMA monomers. (**A**-**B**) SDSPAGE of supernatants (S) and pellets (P) post high-speed centrifugation. RSMA is constant at 2 μM, (**A**) PfPfn and (**B**) PfADF1 are varied from 0-16 μM. (**C**) Quantification by densitometry of the percentage of RSMA remaining in the supernatant. Values are +/- S.E.M (n=3).
