# Supplementary material for: Plasmodium actin is incompletely folded by heterologous protein-folding machinery and likely requires the native Plasmodium chaperonin complex to enter a mature functional state
Source: FASEB J. 2015 Oct 6;30(1):405–16. doi: 10.1096/fj.15-276618 (PMC5423778; doi:10.1096/fj.15-276618)
Supplement: Supplemental Data [file supp_fj.15-276618_Supplemental_Figure2.pdf]

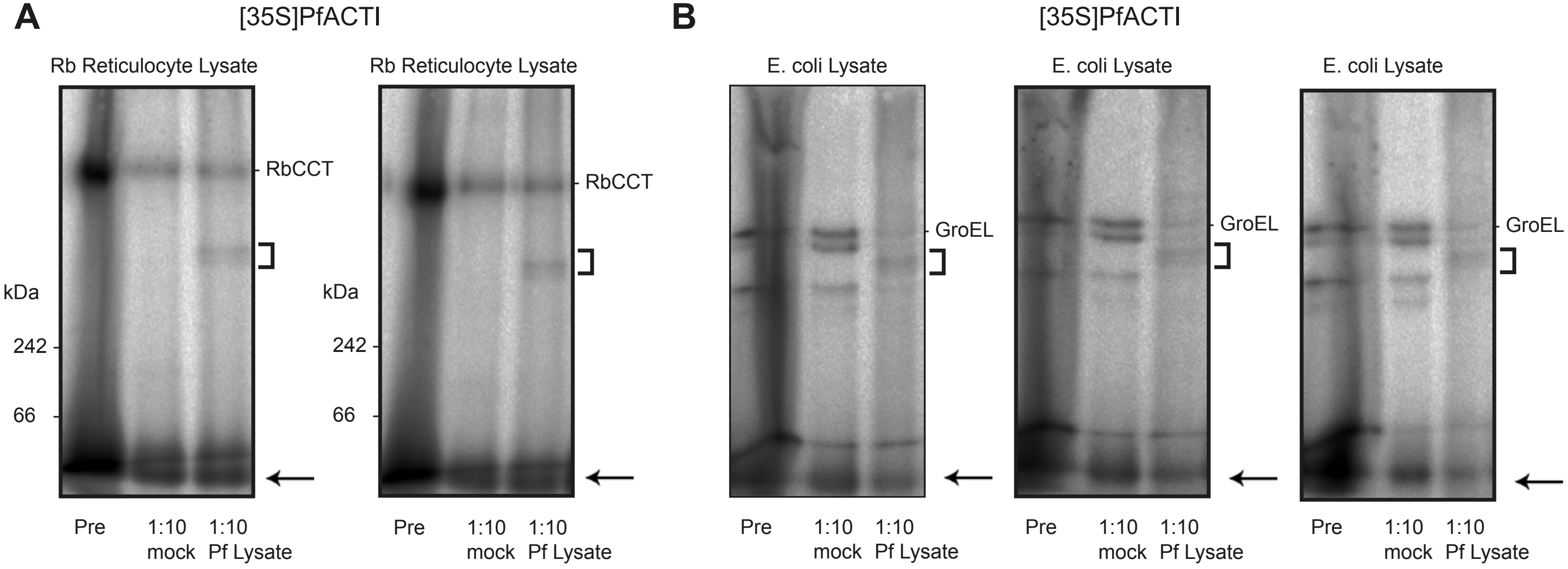

Supplementary Figure 2: Repetition Experiments of [35S]-PfACTI added to *P. falciparum* cell extract. Repeated native PAGE analysis of [35S]-PfACTI translated in (**A**) rabbit reticulocyte (two additional replicates) or (**B**) *E. coli* lysate (three additional replicates) for 40 minutes (Pre), added to extract preparation buffer (mock) or *P. falciparum* cell extract (Pf lysate) at a 1:10 ratio of [35S]-PfACTI:P. falciparum cell extract for 15 min. Square bracket indicates possible complex formation in *P. falciparum* lysate. Arrow indicates monomeric [35S]-PfACTI.
