# Supplementary material for: Plasmodium actin is incompletely folded by heterologous protein-folding machinery and likely requires the native Plasmodium chaperonin complex to enter a mature functional state
Source: FASEB J. 2015 Oct 6;30(1):405–16. doi: 10.1096/fj.15-276618 (PMC5423778; doi:10.1096/fj.15-276618)
Supplement: Supplemental Data [file supp_fj.15-276618_Supplemental_Figure3.pdf]

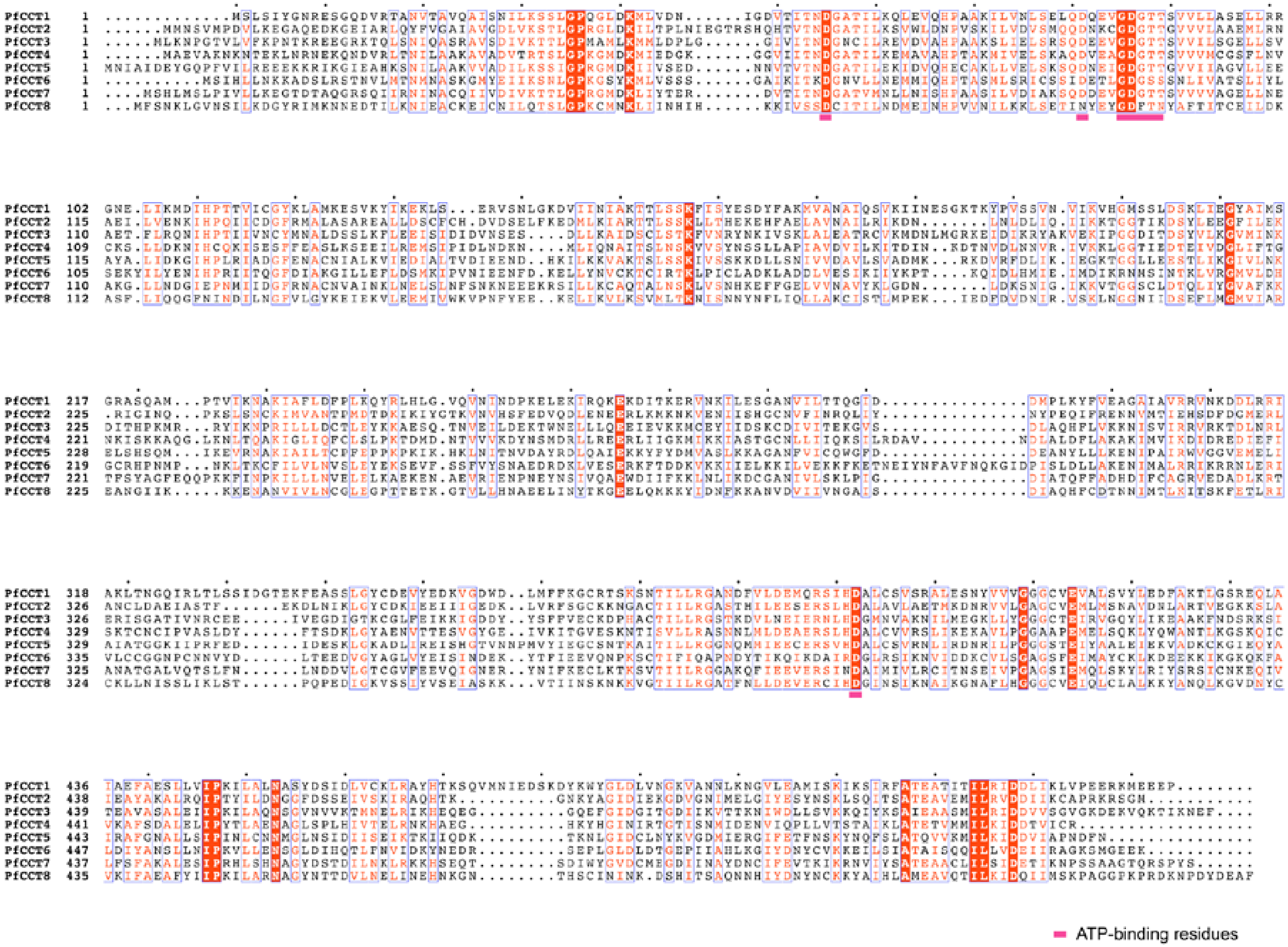

Supplementary Figure 3: Multiple sequence alignments of PfCCT subunits

Multiple sequence alignment of all PfCCT subunits showing conservation between residues. The aspartic acid residues potentially involved in ATP hydrolysis identified across the subunits are highlighted (pink bar) demonstrating likely conservation of the catalytic site.
